# Stage-Dependent Antibiofilm Effects of UVA Combined with Cinnamaldehyde Against *Staphylococcus aureus* Biofilms on Titanium Surfaces

**DOI:** 10.3390/antiox15050574

**Published:** 2026-05-01

**Authors:** Le Wan, Chan-Young Lee, Woochul Jung, Hongyan Zhou, Youzhen Zheng, Kyung-Soon Park

**Affiliations:** 1 Department of Orthopedic Surgery, Center for Joint Disease, Chonnam National University Medical School and Hospital, Hwasun-gun 58128, Republic of Korea; wle202302@gmail.com (L.W.); cnuhoslee@gmail.com (C.-Y.L.); 2617371@gmail.com (W.J.); zyz941207@hotmail.com (Y.Z.); 2 Department of Heart Research Center, Chonnam National University Medical School and Hospital, Gwangju 61469, Republic of Korea; zhouhy202111@gmail.com

**Keywords:** *Staphylococcus aureus*, biofilms, titanium, ultraviolet rays, reactive oxygen species, cinnamaldehyde

## Abstract

*Staphylococcus aureus* biofilms formed on titanium surfaces are highly relevant to orthopedic implant-associated infection and remain difficult to control after maturation. This study aimed to evaluate whether ultraviolet A (UVA, 365 nm) combined with cinnamaldehyde (CA) could improve antibiofilm activity against titanium-associated *S. aureus* biofilms in a stage-resolved *in vitro* model and to examine whether the observed responses were associated with reactive oxygen species (ROS). Early stage (8 h) and 24 h biofilm models were established on total hip arthroplasty (THA)-derived titanium discs. After condition screening, 0.5 mM CA combined with 5 min UVA exposure was selected for subsequent experiments. Biofilm biomass was assessed by crystal violet staining, bacterial viability by live/dead staining and colony-forming unit (CFU) enumeration, ROS-associated fluorescence by dihydroethidium (DHE) imaging, and biofilm-associated gene expression by quantitative real-time PCR (qRT-PCR). Chondrocyte viability was also evaluated under the selected antibiofilm-effective conditions. The combined treatment showed stage-dependent antibiofilm effects, with greater biomass reduction in the 8 h biofilm model and marked impairment of bacterial viability and culturability in both models. ROS-associated fluorescence increased under combined exposure and was partially attenuated by N-acetyl-L-cysteine (NAC) in the 24 h biofilm model. In parallel, CA + UVA was associated with lower expression levels of *clfA*, *icaA*, and *icaD* in the 8 h biofilm model and of *icaA*, *icaB*, and *icaD* in the 24 h biofilm model, with partial NAC attenuation in the latter. Chondrocyte viability was lower in all treatment groups than in the untreated control, although the combined treatment did not show an obvious additional decrease compared with the single-treatment groups. These findings indicate that UVA combined with CA exerts stage-dependent antibiofilm effects in an *in vitro* titanium-associated *S. aureus* biofilm model. The observed ROS-associated responses were consistent with, but do not establish, mechanistic involvement. The current treatment setting also requires further optimization before translational applicability can be more confidently considered.

## 1. Introduction

Orthopedic implant-associated infections remain a major clinical challenge, particularly in periprosthetic joint infection, where bacterial adhesion and subsequent biofilm formation markedly reduce treatment efficacy [1,2,3]. Among the causative pathogens, *Staphylococcus aureus* is especially important because of its strong biofilm-forming ability, persistence on implant materials, and increased tolerance to antimicrobial therapy once mature biofilms are established [1,4]. Titanium and its alloys are widely used in orthopedic devices because of their favorable mechanical properties and biocompatibility; however, these materials can also serve as substrates for bacterial colonization and biofilm development, making antibiofilm control on titanium surfaces highly relevant for translational orthopedic research [5,6,7].

Biofilm development is a dynamic process, and susceptibility to external intervention depends strongly on the developmental stage [8,9]. Early biofilms are generally more vulnerable because attachment is still being established, whereas mature biofilms possess denser cell packing, more structured extracellular matrix, and greater tolerance to antimicrobial or physical treatment [8,9]. Recent studies on clinically relevant orthopedic surfaces have further shown that biofilm maturation is accompanied by marked phenotypic and molecular changes [10,11], supporting the need to distinguish between early-stage and mature biofilms when evaluating new antibiofilm strategies. This distinction is particularly important in implant-related settings, because preventing biofilm establishment and disrupting pre-existing mature biofilms represent biologically different therapeutic goals [1,11].

Cinnamaldehyde (CA), a major bioactive constituent of cinnamon essential oil, has attracted increasing attention as a natural antibacterial and antibiofilm compound [12,13,14]. Previous studies have shown that cinnamaldehyde can interfere with quorum sensing, reduce virulence, suppress biofilm formation, and alter biofilm-associated gene expression in multiple bacterial species, including *Vibrio* spp., *Pseudomonas aeruginosa*, methicillin-resistant *Staphylococcus aureus* (MRSA), and *Staphylococcus epidermidis* [15,16,17,18]. Mechanistically, cinnamaldehyde has been associated with quorum-sensing interference and the modulation of biofilm-related regulatory pathways, including LuxR-related signaling [19], the repression of quorum-sensing-regulated responses in *P. aeruginosa* [17], the inhibition of *staphylococcal* biofilm regulators such as *sarA* [20,21], and downregulation of biofilm-associated genes in MRSA models [16,21]. Collectively, these studies support cinnamaldehyde not only as a direct antibacterial agent, but also as an antivirulence and antibiofilm lead compound [12,13,14].

Ultraviolet irradiation is another promising approach for biofilm control, but its effects depend strongly on wavelength, dose, and biofilm structure [4,22]. Previous studies have shown that UV-based systems can inhibit surface-associated biofilms, reduce the viability of biofilm-embedded bacteria, and, in some settings, prevent biofilm establishment; however, mature biofilms remain more difficult to eradicate because extracellular matrix composition, cell density, and biofilm age can markedly limit UV efficacy [4,23,24]. In this context, UVA (365 nm) is of particular interest. Unlike germicidal ultraviolet C (UVC), UVA is generally less potent as a standalone bactericidal treatment, but it may act as a redox-associated trigger or sensitizing stimulus that enhances the activity of a co-administered antibiofilm agent [25,26]. This feature makes UVA particularly suitable for combination strategies, in which it may potentiate the activity of a chemical antibiofilm agent rather than serve as the primary killing factor itself [26,27].

**Figure 1:**
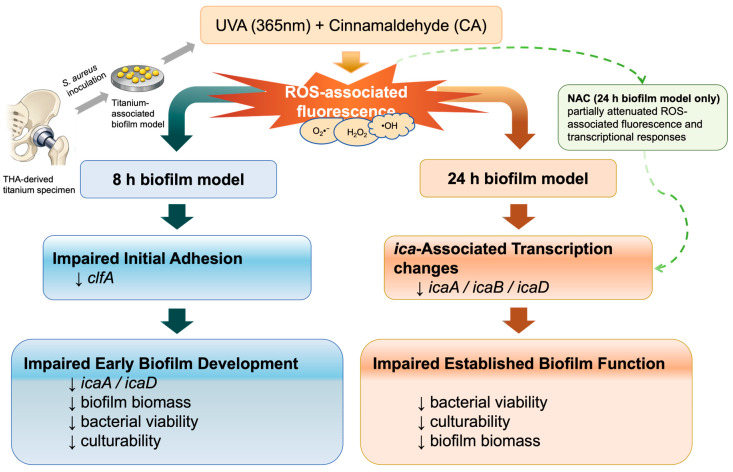
Schematic illustration of the experimental workflow and proposed stage-dependent antibiofilm effects of UVA (365 nm) combined with cinnamaldehyde (CA) against *Staphylococcus aureus* biofilms formed on total hip arthroplasty (THA)-derived titanium discs. In the 8 h biofilm model, the combined treatment was associated with impaired initial adhesion and reduced early biofilm development. In the 24 h biofilm model, the combined treatment was associated with reduced bacterial viability, decreased culturability, and *ica*-associated transcriptional changes. In the 24 h biofilm model, NAC partially attenuated the ROS-associated fluorescence and transcriptional responses observed under combined treatment.

Despite the growing literature on cinnamaldehyde and UV-based biofilm control, studies integrating these two approaches in an orthopedic titanium-surface context remain limited. In particular, stage-resolved evidence is still lacking regarding whether UVA combined with cinnamaldehyde can inhibit *S. aureus* biofilms on titanium surfaces and whether such effects are accompanied by ROS-associated phenotypic and transcriptional responses, especially in the 24 h biofilm model [4,12,13]. Therefore, 8 h and 24 h *S. aureus* biofilm models were established on titanium surfaces, and the antibiofilm effects of UVA combined with cinnamaldehyde were evaluated with emphasis on biofilm biomass, bacterial viability, culturability, ROS-associated fluorescence, transcriptional responses, and chondrocyte viability under the selected treatment conditions (Figure 1). It was hypothesized that combined UVA and cinnamaldehyde treatment would produce stage-dependent antibiofilm effects in this titanium-associated *in vitro* model and would be accompanied by ROS-associated phenotypic and transcriptional responses.

## 2. Materials and Methods

### 2.1. Bacterial Strain, Culture Conditions, and Reagents

The *Staphylococcus aureus* strain used in this study was *S. aureus* KCTC 1621, purchased from Biozoa Biological Supply (Product No. 155067, Seoul, Republic of Korea) and maintained under biosafety level 2 (BSL-2) laboratory conditions. Since the strain was commercially obtained and not isolated from human subjects, no Institutional Review Board (IRB) approval was required. This strain was selected because it showed stable growth and reproducible biofilm formation under the present *in vitro* conditions, as also confirmed in our previous study [28]. It was used as a controlled and reproducible reference model for proof-of-concept evaluation of antibiofilm responses under defined *in vitro* conditions.

Cinnamaldehyde (CA; Sigma-Aldrich, St. Louis, MO, USA; cat. no. W228613-100G-K) was used as the antibiofilm compound. A 1 M CA stock solution was prepared by dissolving 1.26 mL of CA in dimethyl sulfoxide (DMSO; Sigma-Aldrich, St. Louis, MO, USA; cat. no. D2650) to a final volume of 10 mL. A 1 mM working solution was then prepared by diluting 10 μL of the stock solution to 10 mL with tryptic soy broth (TSB; BD, Franklin Lakes, NJ, USA). The final DMSO concentration in all CA-treated and vehicle-control conditions was approximately 0.044% (*v*/*v*). N-acetyl-L-cysteine (NAC; Sigma-Aldrich, St. Louis, MO, USA; cat. no. A9165) was used as a reactive oxygen species (ROS) scavenger. Unless otherwise stated, all other reagents were of analytical grade.

### 2.2. Preparation of Titanium Discs

Titanium alloy discs were obtained from acetabular cup screw holes provided by Lima Corporate (Villanova di San Daniele del Friuli, Italy). The discs were approximately 8 mm in diameter and 1 mm in thickness. Before use, the discs were cleaned by ultrasonication three times for 30 s each, immersed in 75% ethanol for 30 min, and sterilized by autoclaving. The sterilized discs were placed individually into 24-well tissue culture plates (Falcon, Corning Inc., Corning, NY, USA) for subsequent biofilm formation assays.

### 2.3. Establishment of Titanium-Associated 8 h and 24 h Biofilm Models

Sterile titanium discs were placed individually into 24-well tissue culture plates, and 1 mL of *S. aureus* suspension (approximately 1 × 10^6^ CFU/mL) was added to each well. The plates were incubated at 37 °C in 5% CO_2_.

For the 8 h biofilm model, treatment was initiated immediately after inoculation. A total of 0.5 mL of 1 mM CA working solution and 0.5 mL of TSB were added to each treatment well, resulting in a final CA concentration of 0.5 mM. Control wells received an equal volume of DMSO-containing vehicle and TSB corresponding to the CA-treated condition. After 3 h of incubation, the discs were either exposed to UVA for 5 min or kept at room temperature for the same period in the non-irradiated groups. The discs were then incubated for an additional 5 h before analysis, resulting in a total biofilm formation period of 8 h.

For the 24 h biofilm model, inoculated discs were first incubated for 24 h before treatment. The medium was then replaced, and 0.5 mL of 1 mM CA working solution and 0.5 mL of TSB were added to each treatment well, resulting in a final CA concentration of 0.5 mM. After 1 h of incubation, the discs were either exposed to UVA for 5 min or kept at room temperature for the same period in the non-irradiated groups. The discs were then incubated for an additional 2 h before analysis.

For NAC intervention experiments, an additional CA + UVA + NAC group was included only in the 24 h biofilm model. NAC was applied 30 min before UVA exposure at a final concentration of 1 mM.

### 2.4. UVA Irradiation Conditions and Experimental Groups

UVA (365 nm) irradiation was delivered from above using a handheld UV lamp (ENF-240C/FE, Spectro-UV, Farmingdale, NY, USA) to 24-well plates with the lid in place. According to the manufacturer’s specifications, the typical peak UV-A intensity of this model is 300 µW/cm^2^ at a distance of 25 cm. In this study, the distance between the lamp and the top surface of the plate lid was fixed at 5 cm, and the exposure time was 5 min. Samples were exposed under room-temperature conditions for this short irradiation period; however, temperature was not continuously monitored during exposure. In the non-irradiated groups, samples were kept at room temperature for the same period. Because irradiation was delivered through the plate lid and the actual irradiance at the sample surface was not directly measured, UVA exposure is reported here by lamp model, geometry, distance, and exposure time rather than by experimentally verified energy density.

For both the 8 h and 24 h biofilm models, four experimental groups were included: control (Ctrl), CA, UVA, and CA + UVA. In the Ctrl group, biofilms received vehicle treatment containing the same volume of DMSO as in the corresponding CA-treated condition, without UVA exposure. In the CA group, biofilms were treated with CA alone without UVA exposure. In the UVA group, biofilms were exposed to UVA alone without CA treatment. In the CA + UVA group, biofilms were treated with CA followed by UVA exposure under the conditions described above.

For selected experiments in the 24 h biofilm model, an additional CA + UVA + NAC group was included to evaluate ROS-associated involvement. In this group, NAC was applied 30 min before UVA exposure at a final concentration of 1 mM.

### 2.5. Crystal Violet Assay for Biofilm Biomass on Titanium Discs

After treatment, titanium discs were gently washed with phosphate-buffered saline (PBS) to remove non-adherent bacteria. Biofilm biomass remaining on the disc surface was then quantified by crystal violet staining. Briefly, the discs were stained with 0.1% crystal violet for 30 min at room temperature, washed thoroughly to remove excess dye, and air-dried. The bound crystal violet was subsequently solubilized in 95% ethanol, and the absorbance was measured at 570 nm using a microplate reader (Synergy HTX, BioTek, Winooski, VT, USA). Biofilm biomass was expressed as OD570.

### 2.6. Live/Dead Staining of Biofilm-Derived Bacterial Suspensions

Bacterial viability was assessed using the SYTO-9/PI Live/Dead BacLight Bacterial Viability Kit (Fushenbio, Shanghai, China) according to the manufacturer’s instructions. After treatment, titanium discs were gently rinsed with phosphate-buffered saline (PBS) to remove loosely attached bacteria and debris. As the titanium discs were opaque and unsuitable for direct fluorescence imaging of surface-attached biofilms, the biofilm material was recovered from the disc surface for staining.

Briefly, 500 μL of PBS was added to each disc, and the biofilm was carefully scraped from the titanium surface using a sterile scraper. The recovered biofilm material was gently resuspended by pipetting to obtain a biofilm-derived bacterial suspension. An aliquot of the suspension was then stained with the SYTO-9/PI mixture for 15 min at room temperature in the dark. After staining, the samples were placed on glass slides and observed using a Lionheart FX automated microscope (BioTek, Winooski, VT, USA).

Live bacteria were stained green by SYTO-9, whereas dead or membrane-compromised bacteria were stained red by propidium iodide (PI). All imaging experiments were performed in triplicate. Fluorescence images were analyzed using Fiji (ImageJ, version 1.52p; National Institutes of Health, Bethesda, MD, USA). Image acquisition settings were kept constant within each experiment, and quantitative image analysis was performed using the same predefined workflow for all groups within the same experiment. Live and dead bacterial signals were quantified separately, and the percentage of live bacteria was calculated relative to the total bacterial signal.

### 2.7. Enumeration of Culturable Bacteria by Colony-Forming Unit (CFU) Counting

To quantify culturable bacteria within titanium-associated biofilms, treated titanium discs were transferred into sterile tubes containing 500 μL of phosphate-buffered saline (PBS). Biofilms were detached from the disc surface by vortexing for 10 s followed by ultrasonication for 30 s, and this procedure was repeated three times. The resulting suspensions were then serially diluted as appropriate.

Aliquots (50 μL) of the diluted suspensions were plated onto mannitol salt agar (MSA; NutriSelect Basic, Millipore, Darmstadt, Germany) and incubated at 37 °C for 36 h. Colonies were then counted, and the results were expressed as log10 CFU/disc.

### 2.8. Detection of Intracellular ROS

Intracellular ROS-associated fluorescence was assessed using dihydroethidium (DHE; Thermo Fisher Scientific, Waltham, MA, USA; cat. no. D1168) according to the manufacturer’s instructions. After treatment, titanium discs were gently rinsed twice with phosphate-buffered saline (PBS) to remove loosely attached bacteria and debris. As the titanium discs were opaque and unsuitable for direct fluorescence imaging of surface-attached biofilms, the biofilm material was recovered from the disc surface for ROS staining.

Briefly, 500 μL of PBS was added to each disc, and the biofilm was carefully scraped from the titanium surface using a sterile scraper. The recovered biofilm material was gently resuspended by pipetting to obtain a biofilm-derived bacterial suspension. An aliquot of the suspension was then incubated with 5 μM DHE for 15 min at 37 °C in the dark. The samples were washed twice with PBS before staining and washed again with PBS after staining. The stained samples were then placed on glass slides and imaged using a Lionheart FX automated microscope (BioTek, Winooski, VT, USA).

All imaging experiments were performed in triplicate. Fluorescence images were analyzed using Fiji (ImageJ, version 1.52p; National Institutes of Health, Bethesda, MD, USA). Image acquisition settings were kept constant within each experiment, and quantitative image analysis was performed using the same predefined workflow for all groups within the same experiment. Images were converted to 8-bit format, and a uniform threshold was applied to all images within the same experiment to identify fluorescence-positive regions. ROS-associated fluorescence was expressed as the percentage of fluorescence-positive area relative to the total image area.

### 2.9. NAC Intervention Assay

To evaluate ROS-associated responses in the antibiofilm activity of the combined treatment, N-acetyl-L-cysteine (NAC) was applied in selected experiments in the 24 h biofilm model only. An additional CA + UVA + NAC group was included, in which NAC was administered 30 min before UVA exposure at a final concentration of 1 mM.

Following NAC pretreatment, 24 h biofilms were subjected to CA + UVA treatment under the same conditions described above. The effects of NAC were evaluated by live/dead staining, ROS fluorescence analysis, and quantitative real-time PCR. Partial attenuation of CA + UVA-related fluorescence or transcriptional changes by NAC was interpreted as being consistent with ROS-associated involvement.

### 2.10. Quantitative Real-Time PCR Analysis

Total RNA was isolated from biofilm-derived bacterial samples using TRIzol reagent (Invitrogen/Life Technologies, Carlsbad, CA, USA) according to the manufacturer’s instructions. RNA concentration was measured using an ASP-2680 spectrophotometer (ACTGene, Seoul, Republic of Korea). Complementary DNA (cDNA) was synthesized using TOPscript RT DryMIX (Enzynomics, Daejeon, Republic of Korea) in a Mastercycler thermal cycler (Eppendorf, Hamburg, Germany).

Quantitative real-time PCR (qRT-PCR) was subsequently performed using a SYBR Green PCR kit (Enzynomics, Daejeon, Republic of Korea) and gene-specific primers on a Thermal Cycler Dice Real Time System III (TP951; Takara Bio Inc., Shiga, Japan). Primer specificity was assessed by melting-curve analysis. The melting-curve program consisted of 95 °C for 15 s, 60 °C for 1 min, and 95 °C for 15 s with a ramp rate of 0.15 °C/s. Relative gene expression levels were calculated using the 2^−ΔΔCt^ method. The genes analyzed in this study included *clfA*, *icaA*, *icaB*, *icaD*, and *icaR*. The primer sequences used in the present study were the same as those used in our previous study [28], which evaluated the same *Staphylococcus aureus* strain and target gene panel. The *16S* rRNA gene was used as the internal reference for normalization in this targeted qRT-PCR analysis. All primer sequences used in this study are listed in Table 1.

For the 8 h biofilm model, qRT-PCR analysis was performed in the Ctrl, CA, UVA, and CA + UVA groups. For the 24 h biofilm model, an additional CA + UVA + NAC group was included to evaluate whether NAC was associated with partial attenuation of CA + UVA-related transcriptional changes.

### 2.11. Chondrocyte Viability Assay

To provide an initial *in vitro* indication of host–cell compatibility under the selected antibiofilm-effective conditions, human articular chondrocytes (HC-a; ScienCell Research Laboratories, Carlsbad, CA, USA; cat. no. 4650) were cultured in Dulbecco’s Modified Eagle Medium (DMEM; Gibco, Grand Island, NY, USA; REF 11995-065) at 37 °C in a humidified incubator containing 5% CO_2_. When cell confluence reached approximately 80%, the cells were passaged and transferred for further culture. Cells at passages 3–5 were used for the experiments.

Cells were seeded into 96-well plates (Falcon, Corning Inc., Corning, NY, USA) at a density of 5000 cells/well in 100 μL of culture medium and allowed to attach for 48 h. The cells were then assigned to four groups: Ctrl, CA, UVA, and CA + UVA. The treatment conditions were identical to those used in the antibiofilm assays, including a final CA concentration of 0.5 mM, UVA exposure for 5 min, and a total treatment duration of 8 h. The Ctrl group received vehicle treatment containing the same amount of DMSO as the CA-treated groups.

Cell viability was assessed using Cell Counting Kit-8 (CCK-8; Dojindo Laboratories, Kumamoto, Japan; code CK04) according to the manufacturer’s instructions. Briefly, 10 μL of CCK-8 solution was added to each well after treatment, followed by incubation for either 30 min or 2 h. Absorbance was measured at 450 nm using a microplate reader (Synergy HTX, BioTek, Winooski, VT, USA). Cell viability was expressed as a percentage relative to the untreated control group.

### 2.12. Statistical Analysis

Unless otherwise stated, each “*n*” represents an independent biological replicate performed on a separate occasion. For assays involving repeated measurements within the same experiment, technical replicates were averaged first, and the resulting biological replicate means were used for statistical analysis. Data are presented as mean ± standard deviation (SD). The variability shown in the figures reflects variation among independent biological replicates. Statistical analyses and graph preparation were performed using GraphPad Prism 10.3.0 (GraphPad Software, San Diego, CA, USA).

Prior to one-way ANOVA, homogeneity of variance was assessed where applicable using tests such as the Brown–Forsythe and Bartlett’s tests. Comparisons among multiple groups were then performed using one-way analysis of variance (ANOVA) followed by Tukey’s multiple-comparisons test. In addition, two-way ANOVA was performed for the biofilm biomass and CFU datasets, using CA and UVA as fixed factors to evaluate their main effects and the CA × UVA interaction. CFU data were analyzed on the log10-transformed scale and are presented as log10 CFU/disc. A two-sided *p* < 0.05 was considered statistically significant.

As several assays, particularly live/dead staining, ROS fluorescence analysis, and qRT-PCR, were performed with limited biological replication (*n* = 3), these analyses should be interpreted cautiously and were treated as exploratory in terms of mechanistic inference.

## 3. Results

### 3.1. Screening of Appropriate Cinnamaldehyde Concentration and UVA Exposure Conditions

**Figure 2:**
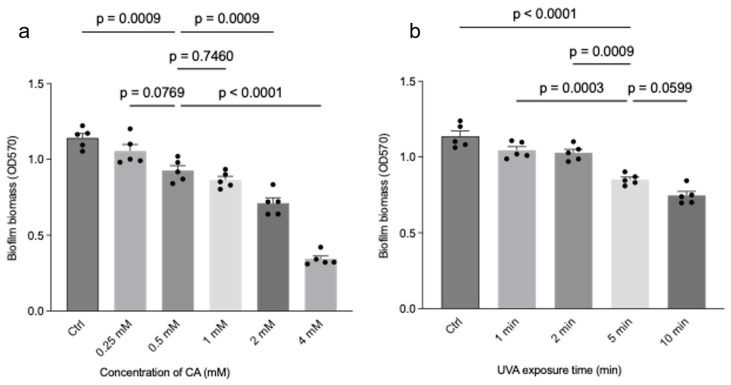
Selection of cinnamaldehyde (CA) and UVA treatment conditions in the 8 h biofilm model. Biofilm biomass on titanium surfaces was quantified by crystal violet staining. OD570 values reflect crystal violet-retained total biofilm biomass after treatment with different CA concentrations and/or UVA exposure conditions. Based on the screening results, a final CA concentration of 0.5 mM and 5 min of UVA exposure were selected for subsequent combination experiments. (**a**) Screening of CA concentration. (**b**) Screening of UVA exposure time. Data are presented as mean ± SD (*n* = 5). Exact *p*-values are shown for the indicated comparisons.

Appropriate treatment conditions for the combination experiments were first determined using the 8 h biofilm model formed on titanium surfaces. Biofilm biomass was quantified by crystal violet staining after exposure to different cinnamaldehyde (CA) concentrations and UVA irradiation conditions. As shown in Figure 2, biofilm biomass progressively decreased as treatment intensity increased. To preserve sufficient dynamic range for evaluating the combined treatment while avoiding near-maximal inhibition by either single treatment alone, a final CA concentration of 0.5 mM and 5 min of UVA exposure were selected for subsequent experiments. Under these conditions, a measurable but non-maximal inhibitory effect was observed in the 8 h biofilm model, indicating that the selected parameters were suitable for subsequent stage-resolved experiments.

### 3.2. Combined Treatment Showed Stage-Dependent Effects on Total Biofilm Biomass

**Figure 3:**
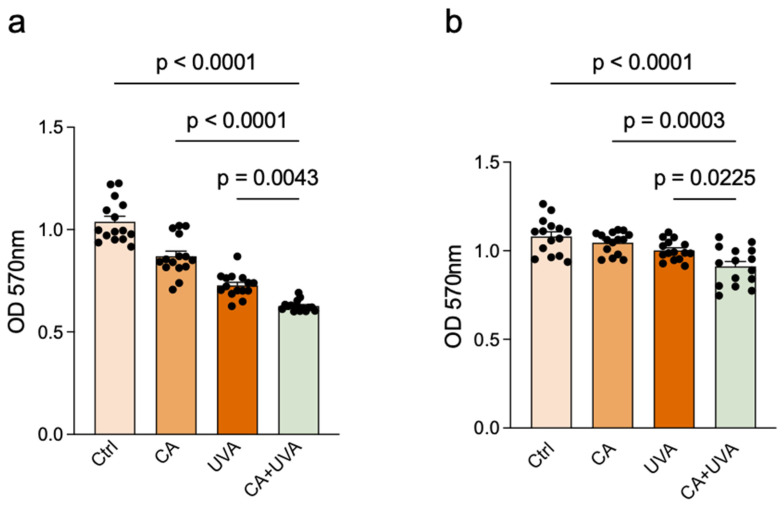
Stage-dependent effects of combined UVA and cinnamaldehyde on titanium-associated *Staphylococcus aureus* biofilm biomass. Biofilm biomass was quantified by crystal violet staining in the 8 h and 24 h biofilm models after treatment with 0.5 mM cinnamaldehyde (CA), 5 min of UVA exposure, or their combination. OD570 values reflect crystal violet-retained total biofilm biomass. The combined treatment reduced biofilm biomass in both models, with a greater reduction in the 8 h model than in the 24 h model. (**a**) 8 h biofilm model. (**b**) 24 h biofilm model. Data are presented as mean ± SD (*n* = 15), and n represents independent biological replicates. Exact *p*-values for pairwise comparisons are shown in the figure. Additional two-way ANOVA showed significant main effects of CA and UVA in the 8 h biofilm model (CA: *p* = 9.92 × 10^−9^; UVA: *p* = 1.04 × 10^−19^), whereas the CA × UVA interaction was not statistically significant (*p* = 0.0941). In the 24 h biofilm model, significant main effects of CA (*p* = 0.0052) and UVA (*p* = 0.000008) were also detected, whereas the CA × UVA interaction was not statistically significant (*p* = 0.2050).

The effect of the selected treatment condition on total biofilm biomass was further evaluated in the 8 h and 24 h titanium-associated biofilm models by crystal violet staining. As shown in Figure 3, CA or UVA alone produced only limited to moderate reductions in biofilm biomass, whereas the combined treatment produced a larger reduction in both models. The magnitude of biomass reduction was greater in the 8 h biofilm model than in the 24 h biofilm model. Additional two-way ANOVA showed significant main effects of both CA and UVA on biofilm biomass in the 8 h and 24 h biofilm models, whereas no significant CA × UVA interaction was detected in either model.

### 3.3. Combined Treatment Primarily Impaired Bacterial Viability and Culturability Within Biofilms

**Figure 4:**
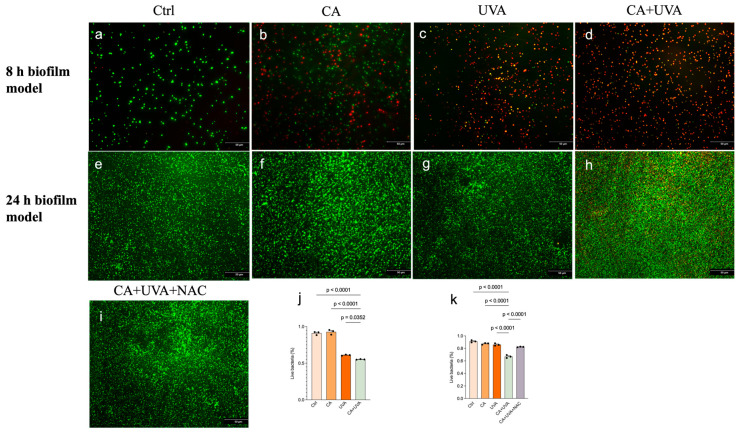
Live/dead staining of biofilm-derived bacterial suspensions recovered from titanium-associated *Staphylococcus aureus* biofilms after treatment with CA, UVA, or their combination. Representative fluorescence images of the 8 h biofilm model (**a**–**d**) and 24 h biofilm model (**e**–**i**) are shown. Green fluorescence indicates live bacteria, whereas red fluorescence indicates membrane-compromised or dead bacteria. Quantitative analysis of the percentage of live bacteria is shown for the 8 h biofilm model (**j**) and 24 h biofilm model (**k**). In the 24 h biofilm model, an additional CA + UVA + NAC group was included to evaluate ROS-associated involvement. The combined treatment reduced the percentage of live bacteria in both models, and this effect was partially attenuated by NAC in the 24 h biofilm model. Data are presented as mean ± SD (*n* = 3). Exact *p*-values are shown for the indicated comparisons. Scale bars = 50 μm.

As crystal violet staining reflects total attached biomass rather than the physiological state of biofilm-embedded bacteria, bacterial viability was further evaluated by live/dead staining. As shown in Figure 4, the percentage of live bacteria was markedly reduced by the combined treatment in both the 8 h and 24 h biofilm models compared with the control and single-treatment groups. In the 8 h biofilm model, UVA alone also reduced the proportion of live bacteria. In the 24 h biofilm model, the combined treatment produced a clearer reduction in the percentage of live bacteria than either single treatment. In addition, partial restoration of the live bacterial proportion was observed after NAC treatment in the 24 h biofilm model.

**Figure 5:**
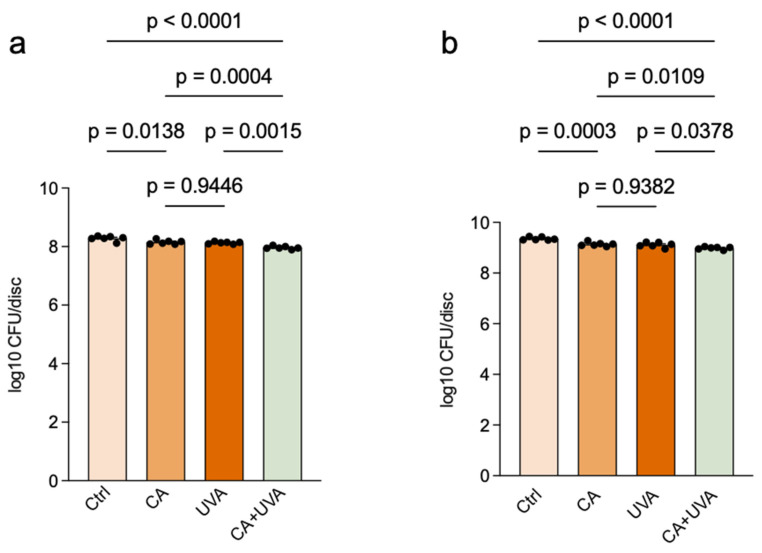
Effects of cinnamaldehyde (CA), UVA, and their combination on culturable bacteria in titanium-associated *Staphylococcus aureus* biofilms. Culturable bacteria were quantified by colony counting and are presented as log10 CFU/disc in the 8 h biofilm model (**a**) and 24 h biofilm model (**b**). The combined treatment reduced culturable bacteria compared with the control and single-treatment groups in both models. Data are presented as mean ± SD (*n* = 6), and n represents independent biological replicates. Exact *p*-values for pairwise comparisons are shown in the figure. CFU data were analyzed on the log10-transformed scale. Additional two-way ANOVA showed significant main effects of CA and UVA in the 8 h biofilm model (CA: *p* = 0.000021; UVA: *p* = 0.000004), whereas the CA × UVA interaction was not statistically significant (*p* = 0.4897). In the 24 h biofilm model, significant main effects of CA (*p* = 8.76 × 10^−7^) and UVA (*p* = 0.000101) were also detected, whereas the CA × UVA interaction was not statistically significant (*p* = 0.5336).

The effect of treatment on biofilm-associated culturable bacteria was further assessed by CFU enumeration. As shown in Figure 5, the combined treatment reduced the number of culturable bacteria in both the 8 h and 24 h biofilm models compared with the control and single-treatment groups. Because CFU data were analyzed on the log10-transformed scale, the results are presented as log10 CFU/disc. Additional two-way ANOVA of the log10-transformed CFU data showed significant main effects of both CA and UVA in the 8 h and 24 h biofilm models, whereas the CA × UVA interaction was not statistically significant in either model.

### 3.4. Combined Treatment Was Associated with Increased ROS-Related Fluorescence, Which Was Partially Attenuated by NAC in the 24 h Biofilm Model

**Figure 6:**
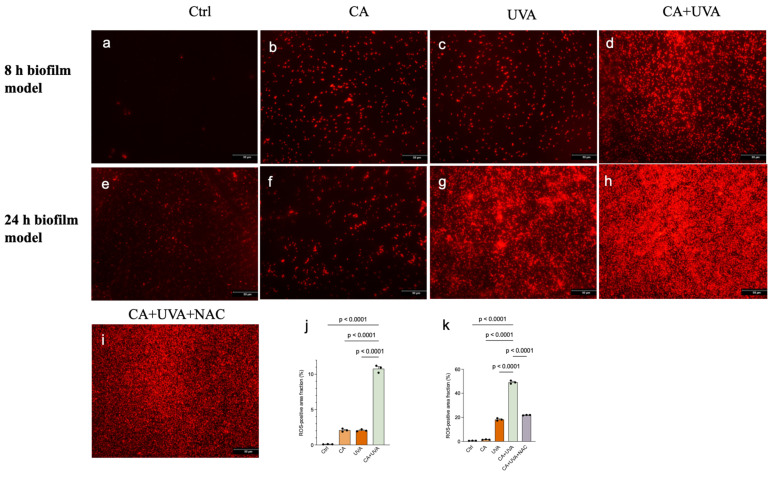
Intracellular ROS-associated fluorescence in titanium-associated *Staphylococcus aureus* biofilms after treatment with cinnamaldehyde (CA), UVA, or their combination. Representative fluorescence images of the 8 h biofilm model (**a**–**d**) and 24 h biofilm model (**e**–**i**) are shown. Quantitative analysis of the fluorescence-positive area fraction is presented for the 8 h biofilm model (**j**) and 24 h biofilm model (**k**). In the 24 h biofilm model, an additional CA + UVA + NAC group was included to evaluate ROS-associated involvement. The combined treatment was associated with an increased fluorescence-positive area fraction in both models, whereas NAC partially attenuated this increase in the 24 h biofilm model. These fluorescence-based findings should be interpreted cautiously and do not establish causality. Data are presented as mean ± SD (*n* = 3). Exact *p*-values are shown for the indicated comparisons. Scale bars = 50 μm.

Intracellular ROS-associated fluorescence was assessed and quantified as the fluorescence-positive area fraction. As shown in Figure 6, ROS-associated fluorescence was higher in the CA + UVA group than in the control and single-treatment groups in both the 8 h and 24 h biofilm models. Quantitative analysis showed that the combined treatment significantly increased the fluorescence-positive area fraction in both models, and this increase was partially attenuated by NAC in the 24 h biofilm model. These image-based data should be interpreted cautiously given the limited biological replication and the indirect nature of fluorescence-based ROS assessment.

### 3.5. Combined Treatment Was Associated with Stage-Dependent Transcriptional Patterns, and NAC Partially Attenuated ica-Associated Changes in the 24 h Biofilm Model

**Figure 7:**
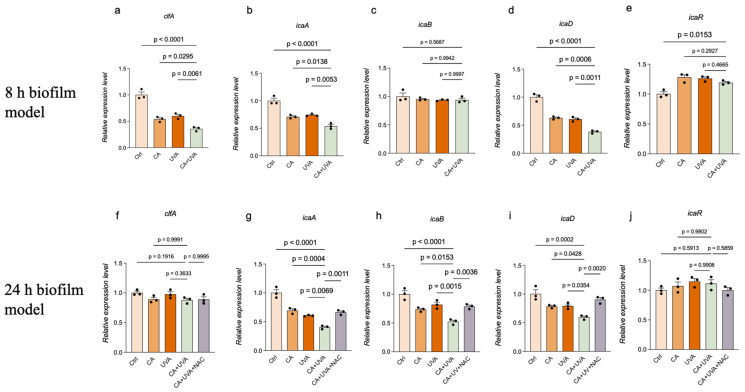
Quantitative real-time PCR analysis of biofilm-associated genes in titanium-associated *Staphylococcus aureus* biofilms after treatment with cinnamaldehyde (CA), UVA, or their combination. Relative expression levels of *clfA*, *icaA*, *icaB*, *icaD*, and *icaR* were analyzed in the 8 h biofilm model (**a**–**e**) and 24 h biofilm model (**f**–**j**). In the 24 h biofilm model, an additional CA + UVA + NAC group was included to evaluate whether NAC partially attenuated CA + UVA-induced transcriptional changes. In the 8 h biofilm model, the combined treatment was associated with lower expression levels of *clfA*, *icaA*, and *icaD*, whereas *icaB* and *icaR* showed limited or no consistent change. In the 24 h biofilm model, the combined treatment was associated with lower expression levels of *icaA*, *icaB*, and *icaD*, and these changes were partially attenuated by NAC. Data are presented as mean ± SD (*n* = 3), and n represents independent biological replicates. These targeted qRT-PCR data should be interpreted cautiously given the limited biological replication. Exact *p*-values are shown for the indicated comparisons.

Molecular responses associated with CA + UVA treatment were further characterized by qRT-PCR analysis of selected biofilm-associated genes in both the 8 h and 24 h biofilm models. In the 8 h biofilm model, the combined treatment was associated with lower expression levels of *clfA*, *icaA*, and *icaD* relative to the control and single-treatment groups, whereas little change was observed in *icaB* and no consistent inhibitory trend was detected for *icaR* (Figure 7).

In contrast, a more focused transcriptional pattern was observed in the 24 h biofilm model. The combined treatment was associated with lower expression levels of *icaA*, *icaB*, and *icaD*, whereas *clfA* and *icaR* showed relatively limited change. In addition, the lower expression levels of *icaA*, *icaB*, and *icaD* observed under combined treatment in the 24 h biofilm model were partially attenuated by NAC. These targeted qRT-PCR findings should be interpreted cautiously given the limited biological replication and the exploratory nature of the gene-expression analysis.

### 3.6. Chondrocyte Viability Under the Selected Antibiofilm-Effective Conditions

**Figure 8:**
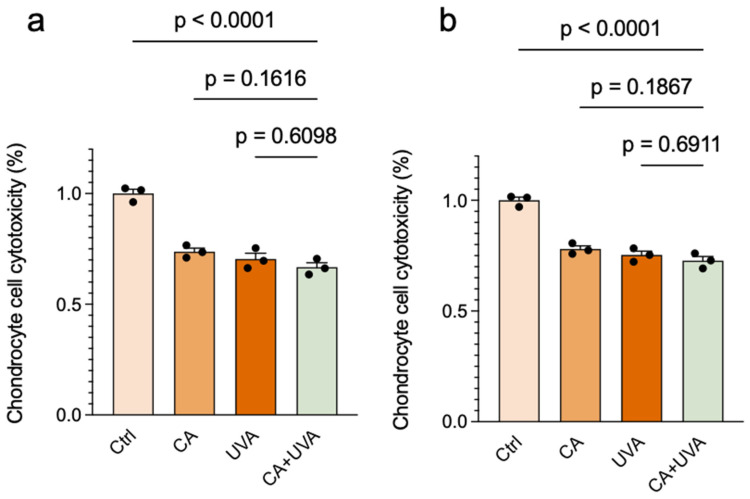
Effects of CA, UVA, and their combination on chondrocyte viability under antibiofilm-effective conditions. Chondrocytes were treated with cinnamaldehyde (CA), UVA, or CA + UVA under the same exposure conditions used in the antibiofilm assays. Cell viability was determined by CCK-8 assay and is expressed as a percentage of the untreated control group. Measurements were obtained after 30 min (**a**) and 2 h (**b**) of CCK-8 incubation. Data are presented as mean ± SD (*n* = 3), with individual data points shown. These results should be interpreted cautiously given the limited biological replication and the absence of a clear additional decrease specific to the combined treatment. Exact *p*-values are shown for the indicated comparisons.

To provide an initial indication of host–cell compatibility under the selected antibiofilm-effective conditions, chondrocytes were exposed to CA, UVA, or their combination, and cell viability was assessed by CCK-8 assay. As shown in Figure 8, chondrocyte viability was lower in all treatment groups than in the untreated control group under both measurement conditions. However, the combined treatment did not show an obvious additional decrease compared with the corresponding single-treatment groups. Given the limited biological replication, these findings should be interpreted cautiously as indicating that the tested exposure setting was not fully cytocompatible relative to the untreated control, rather than as evidence of combination-specific toxicity.

## 4. Discussion

### 4.1. The Combined Treatment Functionally Disabled Biofilms Rather Than Simply Removing Attached Biomass

In the present study, the antibiofilm activity of UVA combined with cinnamaldehyde (CA) against titanium-associated *S. aureus* biofilms was more evident at the level of bacterial viability and culturability than at the level of total biomass. This distinction is important because biofilm biomass and biofilm function are not equivalent endpoints. Crystal violet staining reflects the overall amount of attached material, including bacterial cells, extracellular components, and residual surface-associated structures, whereas live/dead staining and CFU enumeration more directly reflect the physiological state and survival capacity of biofilm-embedded bacteria. In the present study, only a moderate reduction in biomass was detected, whereas clearer reductions were observed in the percentage of live bacteria and in culturable counts. This pattern suggests that the combined treatment primarily compromised the functional integrity of the biofilm by reducing bacterial survival within the attached community rather than by completely removing all attached material.

This interpretation is consistent with the broader cinnamaldehyde literature [12,14]. Cinnamaldehyde has repeatedly been described not only as a direct antibacterial compound, but also as an antivirulence and antibiofilm modulator capable of interfering with quorum sensing, stress adaptation, and biofilm-associated physiology [12,13,14]. In particular, previous work has shown that cinnamaldehyde and its derivatives can reduce biofilm formation and virulence by interfering with quorum-sensing-related signaling, rather than acting solely through immediate biomass eradication [15,17]. Thus, the response pattern observed here is more consistent with the functional disabling of the biofilm than with a simple physical detachment model [21].

It should also be noted that the separation among groups in the CFU assay appeared visually modest when expressed on the log10 scale. However, this should not be interpreted as a weak biological effect. As the CFU data were analyzed on the log10-transformed scale, the differences should be interpreted primarily as lower log10 CFU/disc values rather than as linear percentage changes. When considered together with the live/dead staining results, the CFU data support the interpretation that the combined treatment impaired biofilm-associated bacterial survival, even though only partial biomass reduction was observed. Importantly, two-way ANOVA did not detect a significant CA × UVA interaction for the biomass or CFU endpoints. Therefore, the present findings should be interpreted as greater reductions under combined exposure rather than as evidence of formal synergy. Formal correlation analysis across biomass, viability, and culturability endpoints was not performed in the present study. Such analysis may be valuable in future work to further define the quantitative relationships between structural and functional biofilm responses under combined treatment.

### 4.2. Stage-Dependent Responses Reflected Developmental Differences Between Early and Mature Biofilms

A stage-dependent response to the combined treatment was clearly observed. Greater biomass reduction was detected in the 8 h biofilm model than in the 24 h biofilm model, whereas the 24 h biofilm model remained responsive at the levels of viability, culturability, ROS-associated fluorescence, and transcriptional regulation. This pattern is biologically plausible and should not be interpreted simply as a weaker treatment effect. Rather, it reflects fundamental developmental differences between early-stage and mature biofilms.

During early biofilm development, adhesion and initial intercellular accumulation are still being established [8,9], and structural protection remains relatively limited. By contrast, the 24 h biofilm model was characterized by higher cell density, more consolidated organization, and greater tolerance to external stress [8,9]. This point has also been emphasized in the contemporary UV-biofilm literature [4,22]. Recent reviews have noted that the outcome of UV-based biofilm control is highly dependent on methodology, surface context, and biofilm maturity, and that comparisons across studies are often difficult unless these variables are explicitly considered [22]. In addition, experimental evidence has shown that increased cell density and extracellular matrix composition can significantly reduce biofilm susceptibility to UV treatment [23].

Accordingly, the weaker biomass response observed in the 24 h biofilm model should be interpreted as a manifestation of the intrinsic resilience of mature biofilms rather than as a weakness of the present strategy. Importantly, the persistence of measurable effects in the mature model is of particular relevance, because pre-existing mature biofilms are likely to represent the more clinically challenging phenotype on implant surfaces [1,10,11]. Therefore, the stage-resolved design used in the present study adds both biological and translational value by distinguishing between the inhibition of early biofilm establishment and interference with already developed biofilms.

### 4.3. UVA Showed Limited Standalone Effects and Was Associated with Stronger Responses Under Combined Exposure

The role of UVA in the present system should be interpreted carefully. In the present system, UVA alone produced only limited to moderate antibiofilm effects, whereas much stronger responses were observed when it was combined with CA. At the same time, ROS-associated fluorescence increased under combined exposure, and this increase was partially attenuated by NAC in the 24 h biofilm model. Taken together, these findings indicate that UVA alone had limited effects under the present conditions, whereas stronger responses were observed under combined exposure. However, the current data do not establish a specific ROS-dependent mechanism or a definitive sensitizing role, and these mechanistic interpretations should remain cautious because the present evidence is based primarily on fluorescence-based ROS assessment and partial NAC attenuation rather than on direct pathway dissection.

This interpretation is supported by the broader UV-biofilm literature [4,22]. UV-based biofilm control has increasingly been recognized as highly dependent on wavelength, dose, biofilm architecture, and support surface [4,22]. In many systems, UV efficacy is limited by matrix-associated shielding, surface context, and cell density, which often reduce the effectiveness of irradiation alone and favor combination-based or sensitization-based strategies [22,23]. In parallel, the cinnamaldehyde literature suggests that CA is well suited for such combinational use because its antibacterial potential extends beyond direct growth inhibition to interference with biofilm-associated physiology and regulatory responses [12,13,14]. In addition to ROS-associated oxidative responses, other mechanisms such as membrane perturbation, endogenous photosensitizer-related stress, or non-specific photo-induced damage may also have contributed under UVA exposure.

Therefore, the present data are more consistent with an oxidative stress-associated combination response than with a simple irradiation-only killing model [25,26,27]. In this context, CA may be understood as a bioactive antibacterial and antibiofilm compound evaluated under combined UVA exposure. The partial, rather than complete, attenuation by NAC is noteworthy because it is compatible with ROS-associated involvement, but it does not establish oxidative stress as the sole or definitive mechanism. Likewise, the fluorescence findings should be interpreted as indirect support for ROS-associated responses under combined exposure rather than as pathway-level evidence. However, factorial analysis did not detect a significant CA × UVA interaction for the biomass or CFU endpoints, and therefore the present data do not establish formal synergy.

### 4.4. ROS-Associated Transcriptional Patterns in the 24 h Biofilm Model

The transcriptional findings provide additional context for interpreting the ROS-associated responses observed under combined treatment. In the 8 h biofilm model, lower expression levels of *clfA*, *icaA*, and *icaD* were observed, whereas *icaB* showed little change and *icaR* did not display a consistent inhibitory pattern. This suggests that, in the 8 h biofilm model, the combined treatment was associated primarily with transcriptional changes related to initial adhesion and early biofilm establishment. Such an interpretation is consistent with the known roles of *clfA* in adhesion and of ica-associated genes in early matrix-related accumulation [8,29,30].

In contrast, a more focused transcriptional response was detected in the 24 h biofilm model. Lower expression levels were observed primarily for *icaA*, *icaB*, and *icaD*, whereas *clfA* and *icaR* remained relatively unchanged. This selective pattern may be biologically relevant. Compared with the broader pattern observed in the 8 h biofilm model, this more focused response in the 24 h biofilm model suggests that transcriptional changes under combined treatment differed according to biofilm stage. Rather than indicating the broad, nonspecific repression of all tested biofilm-associated genes, the data suggest that responses in the 24 h biofilm model were associated more prominently with ica-related transcriptional changes [8,9,29]. This point is particularly relevant because the addition of NAC partially attenuated the lower expression levels of *icaA*, *icaB*, and *icaD* in the 24 h biofilm model. This NAC-sensitive pattern is noteworthy because it was observed alongside the ROS-associated fluorescence changes detected in the same model; however, NAC-based attenuation should be interpreted as indirect support for ROS-associated involvement rather than as definitive mechanistic evidence, and the present data remain correlational.

This interpretation is also consistent with previous studies showing that cinnamaldehyde can modulate biofilm-associated regulatory pathways rather than functioning only as a direct bactericidal agent [12,20,21]. Cinnamaldehyde has been reported to interfere with quorum sensing and to alter virulence-related and biofilm-related regulation in different bacterial systems [15,17,21]. Within this broader framework, the present study adds to the discussion by showing that, in titanium-associated *S. aureus* biofilms, combined treatment was accompanied by selective *ica*-associated transcriptional changes in the 24 h biofilm model, alongside ROS-associated fluorescence responses. The lack of a strong *icaR* response should not be interpreted as a weakness; rather, it may indicate that the observed transcriptional effects were selective and pathway-focused rather than uniformly distributed across all tested regulators. However, these transcriptional findings should be interpreted cautiously because the qRT-PCR analysis was based on a limited number of biological replicates and was intended as a targeted exploratory assessment rather than a comprehensive mechanistic analysis.

### 4.5. Translational Relevance and Realistic Boundaries of the Present Model

The translational relevance of the present work is strengthened by the use of THA-derived titanium discs rather than conventional plastic-only biofilm assays. Implant-associated biofilms are well known to be influenced by the physicochemical characteristics of the support surface [5,6,7], and this point has also been emphasized in recent UV-biofilm methodological reviews [4,22], in which surface context has been identified as an important but often underappreciated determinant of treatment outcome. By incorporating a titanium-based model, the present study was able to evaluate the combined treatment in a setting more relevant to orthopedic implant-associated infection [1,10,11].

At the same time, the present model also defines important translational boundaries. A particularly important limitation of the current treatment setting is that chondrocyte viability was lower in all treatment groups than in the untreated control under the selected antibiofilm-effective conditions, indicating limited host–cell compatibility *in vitro*. However, the combined treatment did not appear to produce an obvious additional reduction compared with the corresponding single-treatment groups. Therefore, the present findings should not be interpreted as indicating that CA + UVA uniquely exacerbated host–cell damage, but rather that the current treatment setting as a whole still requires optimization before translational applicability can be more confidently considered. At the present stage, the findings should not be interpreted as establishing a clinically implementable orthopedic treatment protocol, because practical application would require the further optimization of delivery conditions, broader cytocompatibility testing, and additional safety evaluation. In addition, the cytocompatibility experiment was based on limited biological replication and should therefore be regarded as an initial *in vitro* compatibility screen rather than a definitive assessment of host–cell safety. Further optimization of dose/exposure conditions and broader cytocompatibility testing in additional clinically relevant cell types will be necessary before translational feasibility can be more rigorously evaluated. Moreover, although cinnamaldehyde has known irritant potential, irritation-related safety was not evaluated in the present study and will require dedicated assessment in future work. Moreover, although the combined treatment reduced culturable bacteria under the present conditions, the magnitude of reduction remained partial and should be interpreted as proof-of-concept rather than as evidence of immediate clinical applicability.

Several additional limitations should be acknowledged. First, the present work was performed in a controlled *in vitro* system using a single bacterial strain and static exposure conditions, which limits direct generalizability to the complexity of orthopedic implant-associated infections. Future studies should include clinical isolates and resistant strains, including MRSA, to better assess the robustness and translational relevance of the observed responses. In particular, the model did not incorporate polymicrobial biofilms, which are frequently encountered in clinically challenging implant-associated infections and may show different structural and treatment-response characteristics. Second, several assays, particularly the image-based ROS analysis and the targeted qRT-PCR experiments, were performed with limited biological replication, which restricts statistical power and supports the cautious interpretation of these findings. Third, although the data were consistent with ROS-associated involvement, the redox pathway was not dissected beyond fluorescence-based ROS assessment and NAC intervention. DHE-based fluorescence does not provide a highly specific measure of individual oxidative species, NAC may exert biological effects beyond ROS scavenging, and fluorescence area analysis may be influenced by thresholding and imaging variability. Therefore, mechanistic causality cannot be established from the present data alone. Fourth, the 24 h biofilm model used in the present study should not be interpreted as fully representing the complexity of long-established implant-associated biofilms in vivo. Fifth, live/dead staining was performed on biofilm-derived suspensions recovered from the titanium surface rather than on intact surface-associated biofilms, which may limit the interpretation of viability within the native biofilm architecture. Finally, the combined treatment appeared to impair biofilm-associated survival more strongly than it reduced total attached biomass, suggesting that some residual matrix-associated material may remain on the titanium surface after treatment. In addition, although biomass, live/dead staining, and CFU assays were evaluated in parallel, formal integrative analyses across these endpoints were not performed and should be considered in future studies. Formal randomization and blinded image scoring were not implemented in this study. In addition to ROS-associated oxidative responses, other mechanisms such as membrane perturbation, endogenous photosensitizer-related stress, or non-specific photo-induced damage may also have contributed under UVA exposure.

## 5. Conclusions

In conclusion, UVA combined with cinnamaldehyde exerted stage-dependent antibiofilm effects against *Staphylococcus aureus* biofilms formed on titanium surfaces in an *in vitro* model. The combined treatment produced greater effects on bacterial viability and culturability than on total biofilm biomass, indicating that its principal action was more consistent with the functional impairment of biofilm-embedded bacteria than with the complete removal of attached biomass. The observed increase in ROS-associated fluorescence and the partial attenuation by NAC were consistent with ROS-associated involvement, particularly in the 24 h biofilm model; however, the mechanistic evidence remains indirect and does not establish causality. In addition, two-way ANOVA did not demonstrate a significant CA × UVA interaction for the biomass or CFU endpoints. The current exposure setting was also associated with reduced chondrocyte viability relative to the untreated control, which represents an important limitation and indicates that substantial optimization will be required before translational relevance can be more confidently considered. Accordingly, the present findings should be interpreted as proof-of-concept evidence of stage-dependent *in vitro* antibiofilm activity under a defined experimental setting, rather than as support for direct therapeutic or clinical applicability.

## Figures and Tables

**Table 1:** Primer sequences used for quantitative real-time PCR.

**Gene**	**Sequence**
*icaA*	F: CTATTTCGGGTGTCTTCACTC
	R: GGCAAGCGGTTCATACTTA
*icaB*	F: TTGCCTGTAAGCACACTGGATGGTC
	R: TACACGGTGATAATTTAATGCCAGAGC
*icaD*	F: ATGGACAAGTCCAGACAGAGGAAAA
	R: GTCACTCATCGTAACTGCTTCAACG
*icaR*	F: TCAGAGAAGGGGTATGACGGTACAA
	R: TCCTCAGGCGTATTAGATAATTGAACG
*clfA*	F: CAAGTAGCGTTAGTGCTGC
	R: TGATTGAGTTGTTGCCG
*16S rRNA*	F: CGTGCTACAATGGACAATACAAA
	R: ATCTACGATTACTAGCGATTCCA

## Data Availability

The original contributions presented in this study are included in the article. Further inquiries can be directed to the corresponding author.
